# Supplementary material for: A microbial signature following bariatric surgery is robustly consistent across multiple cohorts
Source: Gut Microbes. 2021 Jun 23;13(1):1930872. doi: 10.1080/19490976.2021.1930872 (PMC8224199; doi:10.1080/19490976.2021.1930872)
Supplement: Supplemental Material [file KGMI_A_1930872_SM8444.zip › supplementary/FIGURE 2.pdf]

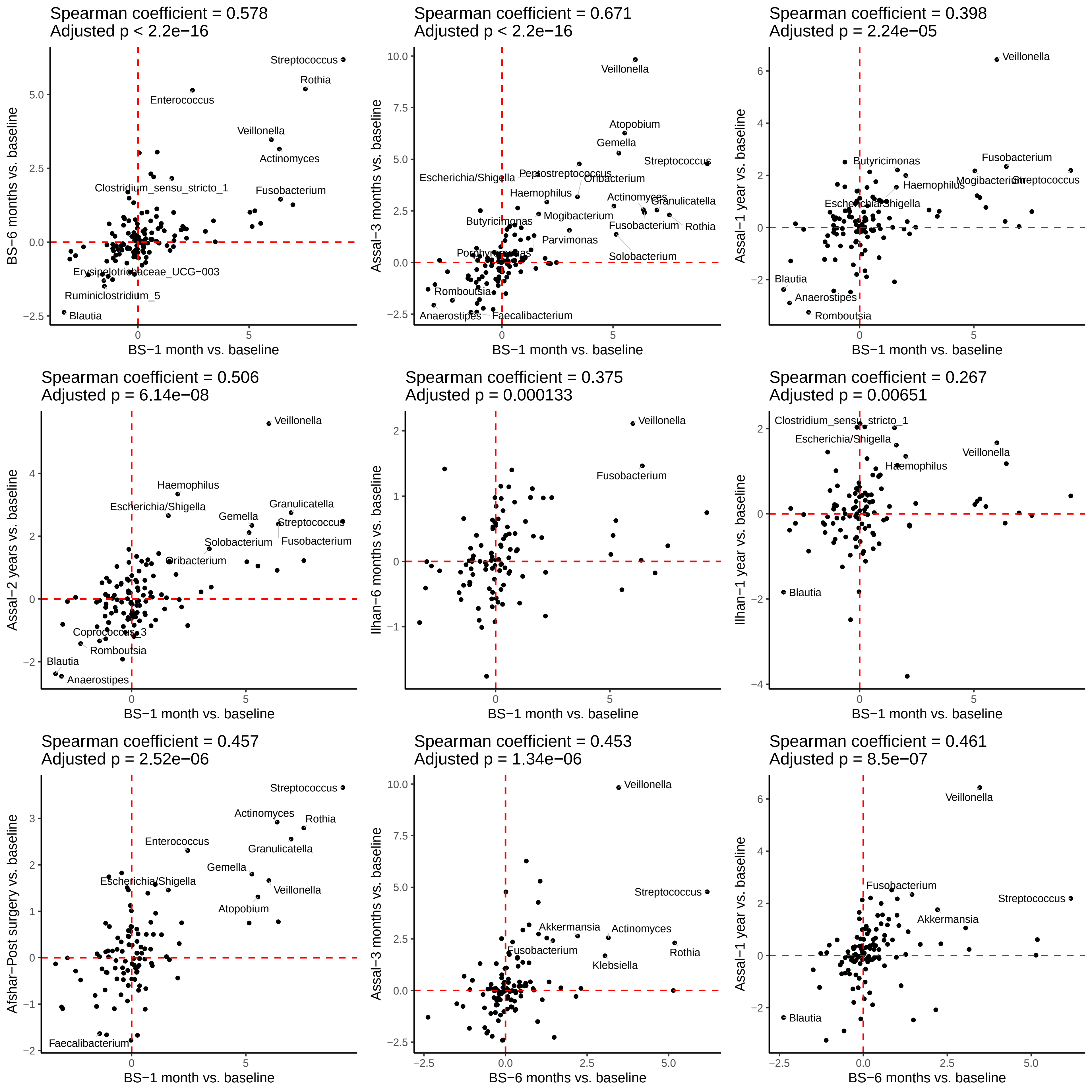

Spearman coefficient = 0.578
Adjusted p < 2.2e−16
Streptococcus
Rothia
Enterococcus
Veillonella
Actinomyces
Fusobacterium
Clostridium_sensu_stricto_1
Erysipelotrichaceae_UCG−003
Ruminiclostridium_5
Blautia
BS−6 months vs. baseline
BS−1 month vs. baseline
Spearman coefficient = 0.671
Adjusted p < 2.2e−16
Veillonella
Atopobium
Gemella
Streptococcus
Escherichia/Shigella
Peptostreptococcus
Oribacterium
Haemophilus
Actinomyces
Granulicatella
Mogibacterium
Butyricimonas
Fusobacterium
Rothia
Parvimonas
Solobacterium
Porphyromonas
Romboutsia
Anaerostipes
Faecalibacterium
Assal−3 months vs. baseline
BS−1 month vs. baseline
Spearman coefficient = 0.398
Adjusted p = 2.24e−05
Veillonella
Butyricimonas
Fusobacterium
Haemophilus
Mogibacterium
Streptococcus
Escherichia/Shigella
Blautia
Anaerostipes
Romboutsia
Assal−1 year vs. baseline
BS−1 month vs. baseline
Spearman coefficient = 0.506
Adjusted p = 6.14e−08
Veillonella
Haemophilus
Escherichia/Shigella
Granulicatella
Gemella
Streptococcus
Solobacterium
Fusobacterium
Oribacterium
Coprococcus_3
Romboutsia
Blautia
Anaerostipes
Assal−2 years vs. baseline
BS−1 month vs. baseline
Spearman coefficient = 0.375
Adjusted p = 0.000133
Veillonella
Fusobacterium
Ilhan−6 months vs. baseline
BS−1 month vs. baseline
Spearman coefficient = 0.267
Adjusted p = 0.00651
Clostridium_sensu_stricto_1
Escherichia/Shigella
Veillonella
Haemophilus
Blautia
Ilhan−1 year vs. baseline
BS−1 month vs. baseline
Spearman coefficient = 0.457
Adjusted p = 2.52e−06
Streptococcus
Actinomyces
Rothia
Enterococcus
Granulicatella
Gemella
Escherichia/Shigella
Veillonella
Atopobium
Faecalibacterium
Afshar−Post surgery vs. baseline
BS−1 month vs. baseline
Spearman coefficient = 0.453
Adjusted p = 1.34e−06
Veillonella
Streptococcus
Akkermansia
Actinomyces
Fusobacterium
Rothia
Klebsiella
Assal−3 months vs. baseline
BS−6 months vs. baseline
Spearman coefficient = 0.461
Adjusted p = 8.5e−07
Veillonella
Fusobacterium
Streptococcus
Akkermansia
Blautia
Assal−1 year vs. baseline
BS−6 months vs. baseline

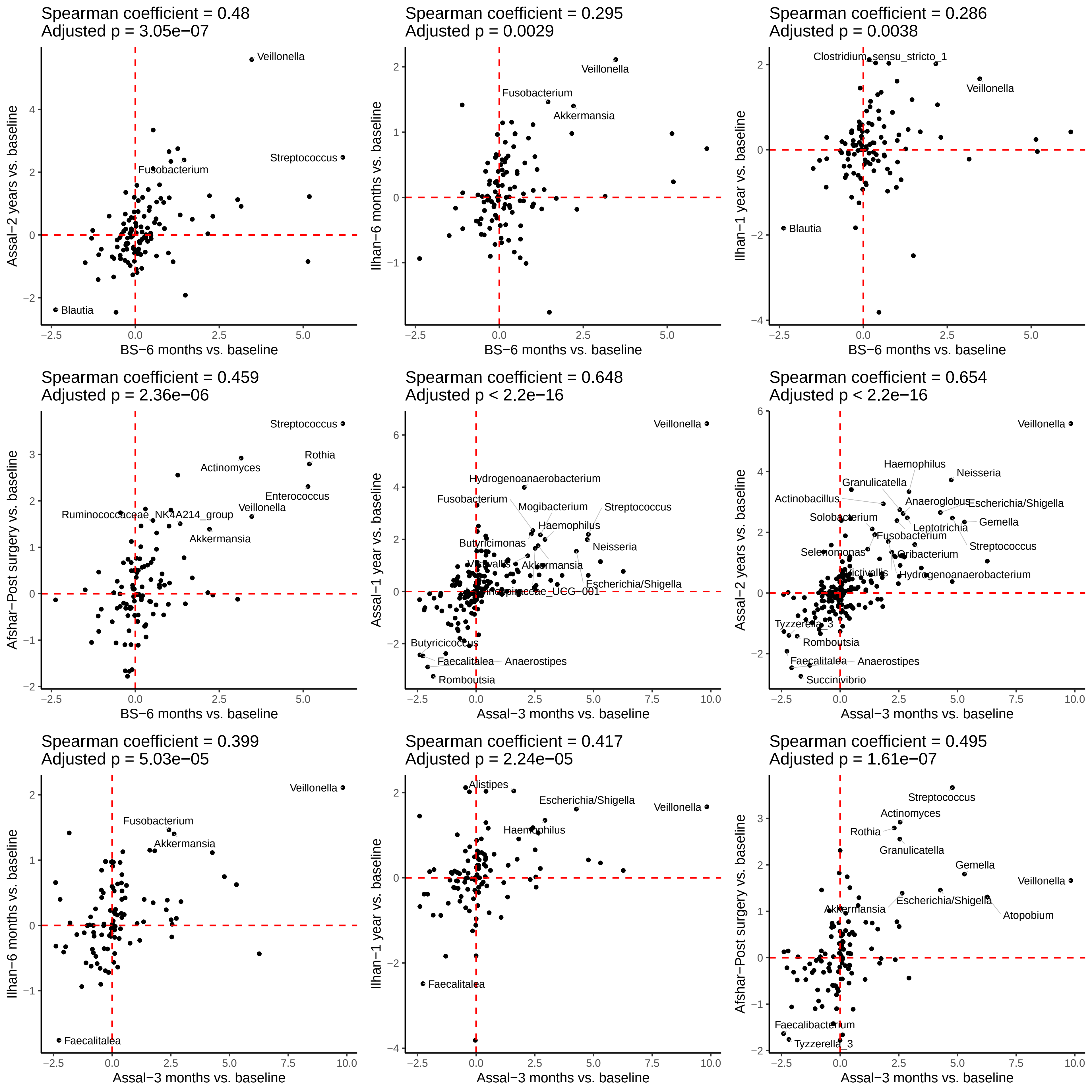
Spearman coefficient = 0.48
Adjusted p = 3.05e−07
Assal−2 years vs. baseline
BS−6 months vs. baseline
Veillonella
Streptococcus
Fusobacterium
Blautia
Spearman coefficient = 0.295
Adjusted p = 0.0029
Ilhan−6 months vs. baseline
BS−6 months vs. baseline
Veillonella
Fusobacterium
Akkermansia
Spearman coefficient = 0.286
Adjusted p = 0.0038
Ilhan−1 year vs. baseline
BS−6 months vs. baseline
Clostridium_sensu_stricto_1
Veillonella
Blautia
Spearman coefficient = 0.459
Adjusted p = 2.36e−06
Afshar−Post surgery vs. baseline
BS−6 months vs. baseline
Streptococcus
Rothia
Actinomyces
Enterococcus
Veillonella
Ruminococcaceae_NK4A214_group
Akkermansia
Spearman coefficient = 0.648
Adjusted p < 2.2e−16
Assal−1 year vs. baseline
Assal−3 months vs. baseline
Veillonella
Hydrogenoanaerobacterium
Fusobacterium
Mogibacterium
Streptococcus
Haemophilus
Butyricimonas
Neisseria
Victivallis
Akkermansia
Escherichia/Shigella
Lachnospiraceae_UCG−001
Butyricicoccus
Faecalitalea
Anaerostipes
Romboutsia
Spearman coefficient = 0.654
Adjusted p < 2.2e−16
Assal−2 years vs. baseline
Assal−3 months vs. baseline
Veillonella
Haemophilus
Neisseria
Granulicatella
Actinobacillus
Anaeroglobus
Escherichia/Shigella
Solobacterium
Leptotrichia
Gemella
Fusobacterium
Streptococcus
Selenomonas
Oribacterium
Victivallis
Hydrogenoanaerobacterium
Tyzzerella_3
Romboutsia
Faecalitalea
Anaerostipes
Succinivibrio
Spearman coefficient = 0.399
Adjusted p = 5.03e−05
Ilhan−6 months vs. baseline
Assal−3 months vs. baseline
Veillonella
Fusobacterium
Akkermansia
Faecalitalea
Spearman coefficient = 0.417
Adjusted p = 2.24e−05
Ilhan−1 year vs. baseline
Assal−3 months vs. baseline
Alistipes
Escherichia/Shigella
Veillonella
Haemophilus
Faecalitalea
Spearman coefficient = 0.495
Adjusted p = 1.61e−07
Afshar−Post surgery vs. baseline
Assal−3 months vs. baseline
Streptococcus
Actinomyces
Rothia
Granulicatella
Gemella
Veillonella
Escherichia/Shigella
Akkermansia
Atopobium
Faecalibacterium
Tyzzerella_3

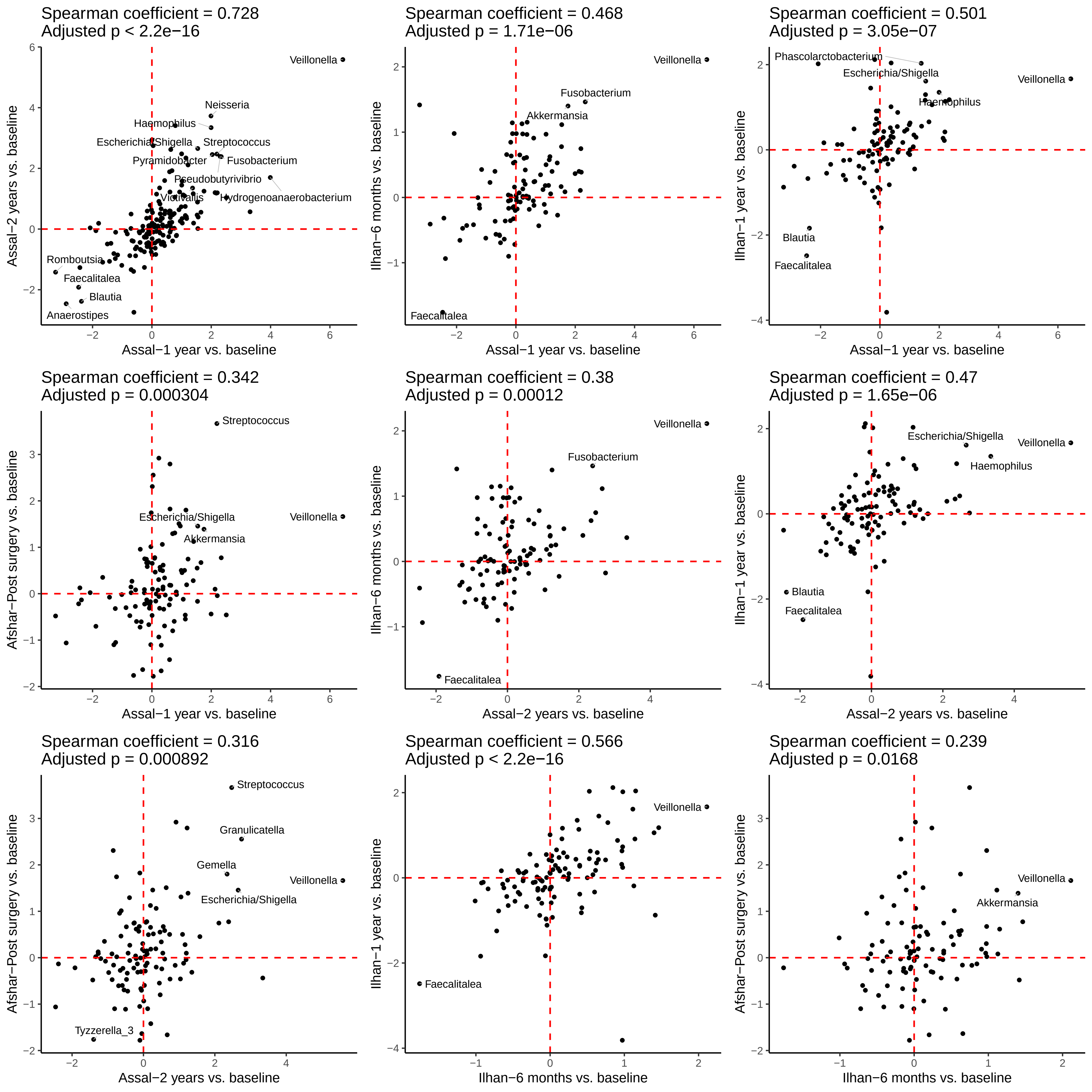
Spearman coefficient = 0.728
Adjusted p < 2.2e−16
Veillonella
Neisseria
Haemophilus
Escherichia/Shigella
Streptococcus
Pyramidobacter
Fusobacterium
Pseudobutyrivibrio
Victivallis
Hydrogenoanaerobacterium
Romboutsia
Faecalitalea
Blautia
Anaerostipes
Assal−2 years vs. baseline
Assal−1 year vs. baseline
Spearman coefficient = 0.468
Adjusted p = 1.71e−06
Veillonella
Fusobacterium
Akkermansia
Faecalitalea
Ilhan−6 months vs. baseline
Assal−1 year vs. baseline
Spearman coefficient = 0.501
Adjusted p = 3.05e−07
Phascolarctobacterium
Escherichia/Shigella
Veillonella
Haemophilus
Blautia
Faecalitalea
Ilhan−1 year vs. baseline
Assal−1 year vs. baseline
Spearman coefficient = 0.342
Adjusted p = 0.000304
Streptococcus
Escherichia/Shigella
Veillonella
Akkermansia
Afshar−Post surgery vs. baseline
Assal−1 year vs. baseline
Spearman coefficient = 0.38
Adjusted p = 0.00012
Veillonella
Fusobacterium
Faecalitalea
Ilhan−6 months vs. baseline
Assal−2 years vs. baseline
Spearman coefficient = 0.47
Adjusted p = 1.65e−06
Escherichia/Shigella
Veillonella
Haemophilus
Blautia
Faecalitalea
Ilhan−1 year vs. baseline
Assal−2 years vs. baseline
Spearman coefficient = 0.316
Adjusted p = 0.000892
Streptococcus
Granulicatella
Gemella
Veillonella
Escherichia/Shigella
Tyzzerella_3
Afshar−Post surgery vs. baseline
Assal−2 years vs. baseline
Spearman coefficient = 0.566
Adjusted p < 2.2e−16
Veillonella
Faecalitalea
Ilhan−1 year vs. baseline
Ilhan−6 months vs. baseline
Spearman coefficient = 0.239
Adjusted p = 0.0168
Veillonella
Akkermansia
Afshar−Post surgery vs. baseline
Ilhan−6 months vs. baseline

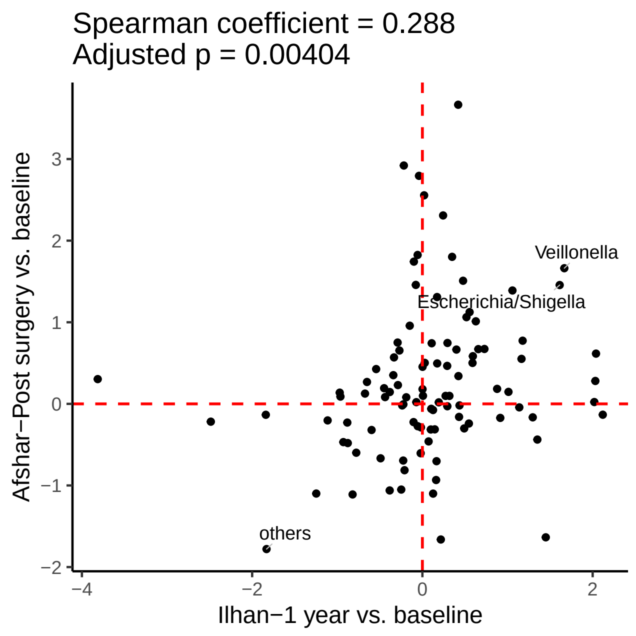
Spearman coefficient = 0.288
Adjusted p = 0.00404
Afshar−Post surgery vs. baseline
Ilhan−1 year vs. baseline
Veillonella
Escherichia/Shigella
others
3
2
1
0
−1
−2
−4
−2
0
2
