# Supplementary figures and images for: A microbial signature following bariatric surgery is robustly consistent across multiple cohorts
Source: Gut Microbes. 2021 Jun 23;13(1):1930872. doi: 10.1080/19490976.2021.1930872 (PMC8224199; doi:10.1080/19490976.2021.1930872)

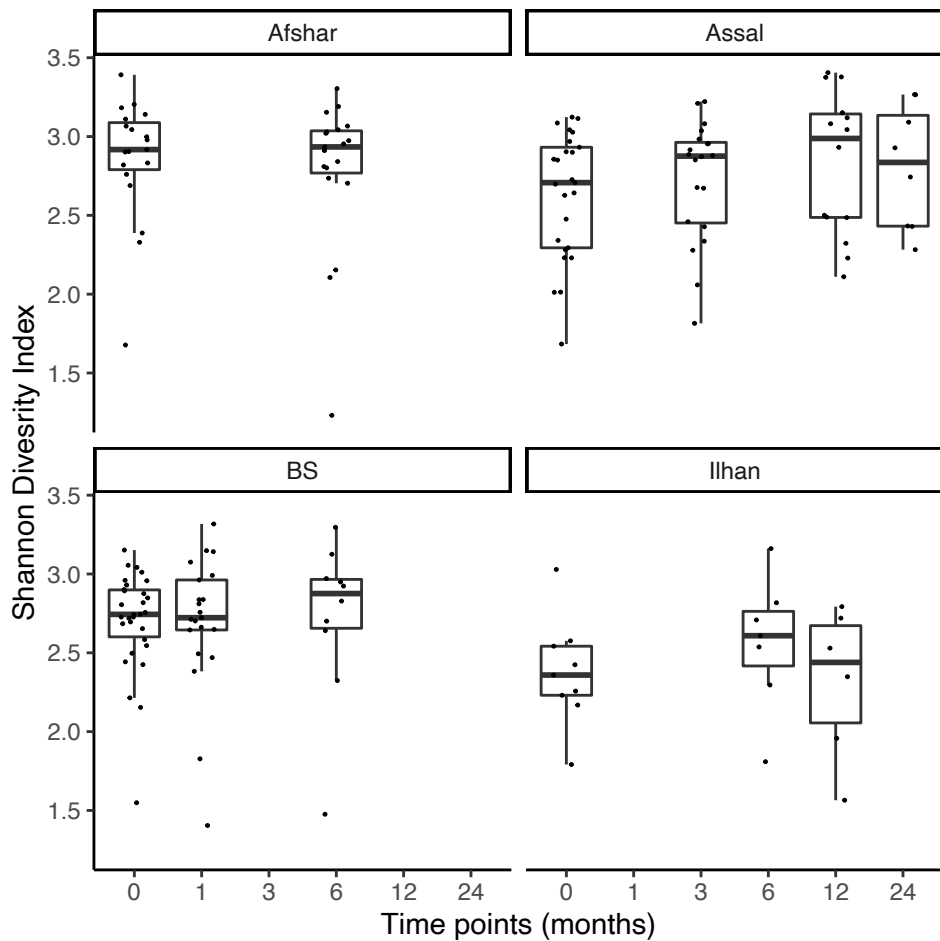

Supplement: Supplemental Material [file KGMI_A_1930872_SM8444.zip › supplementary/FIGURE 1.pdf]

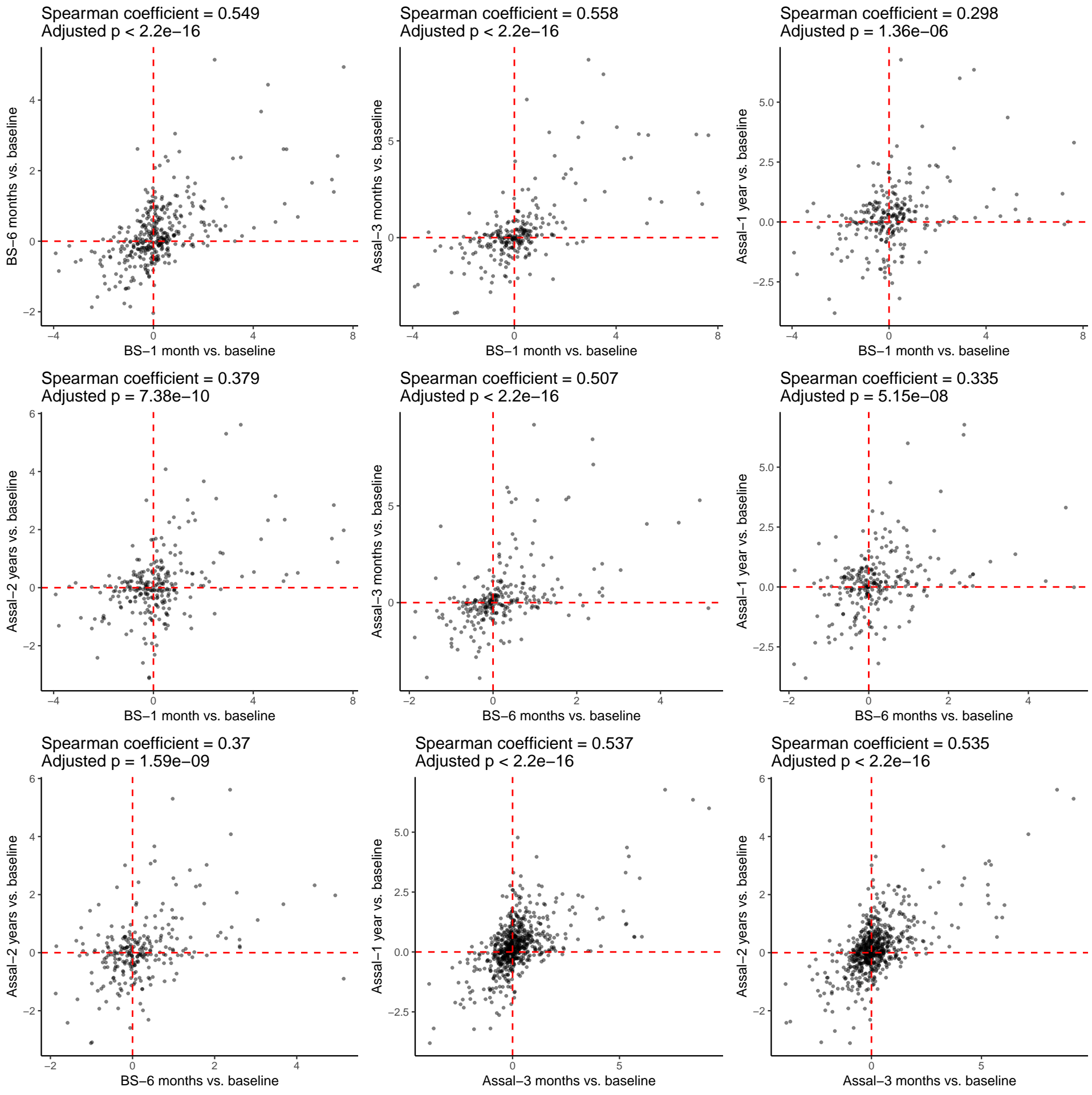

Spearman coefficient = 0.676  
Adjusted p < 2.2e-16

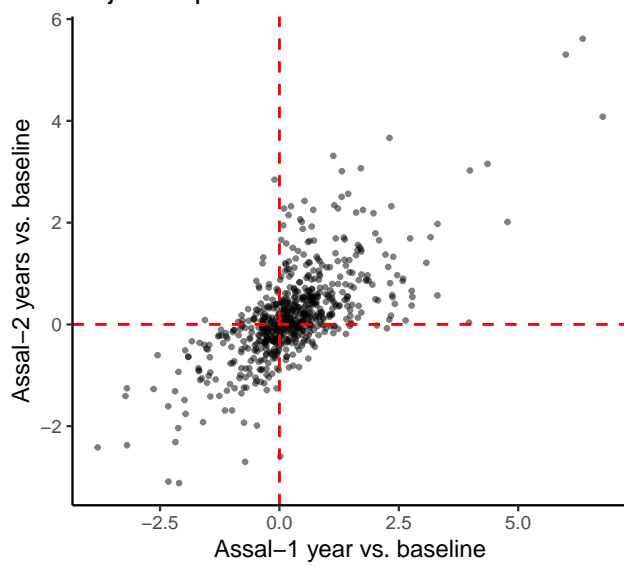

Supplement: Supplemental Material [file KGMI_A_1930872_SM8444.zip › supplementary/FIGURE 3.pdf]

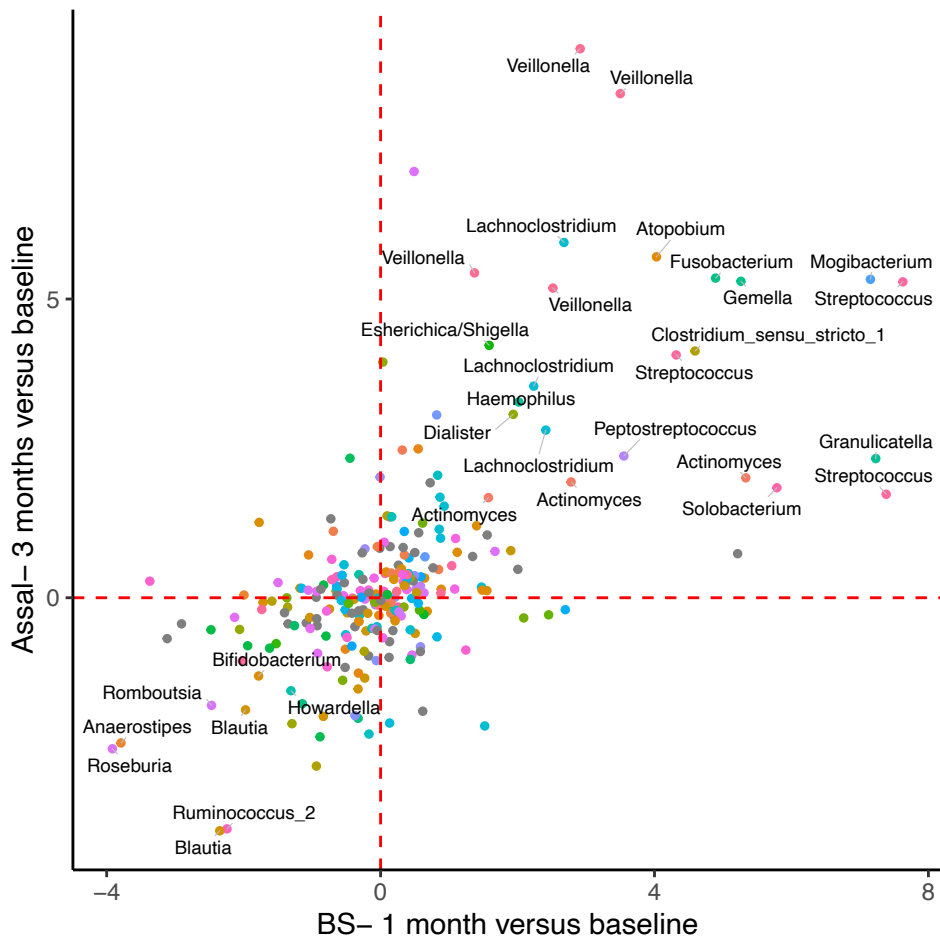

Supplement: Supplemental Material [file KGMI_A_1930872_SM8444.zip › supplementary/FIGURE 4.pdf]

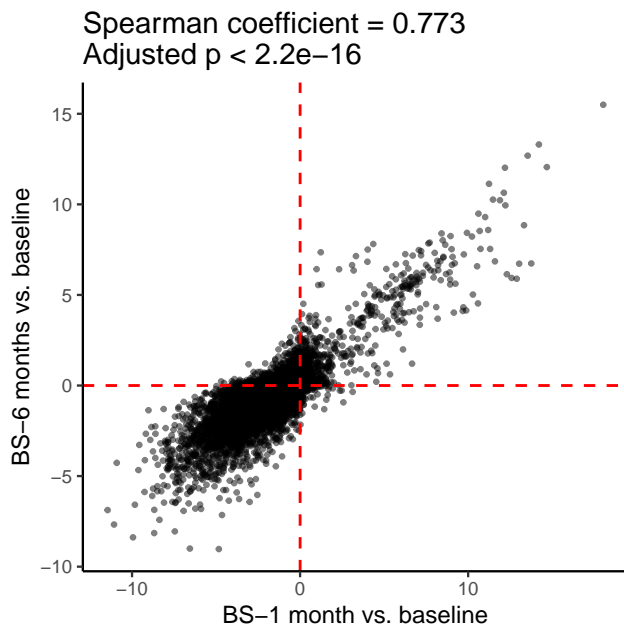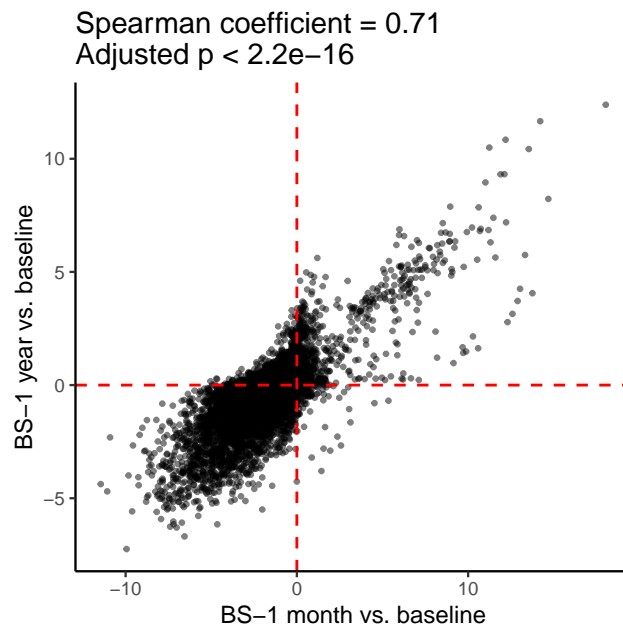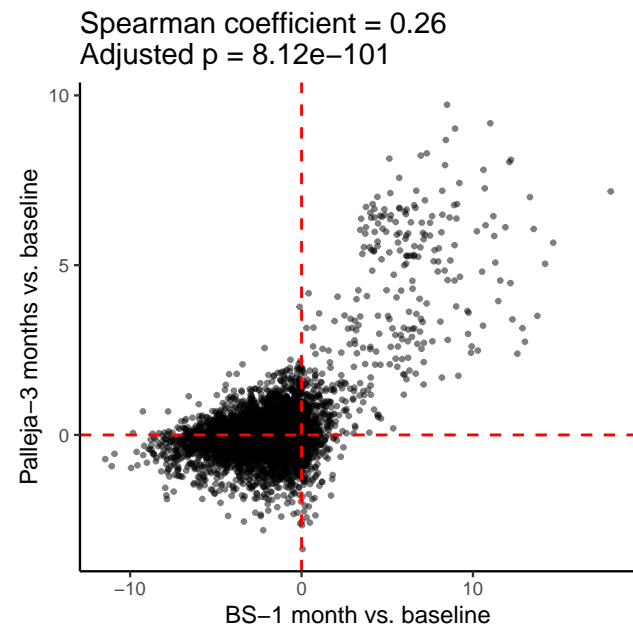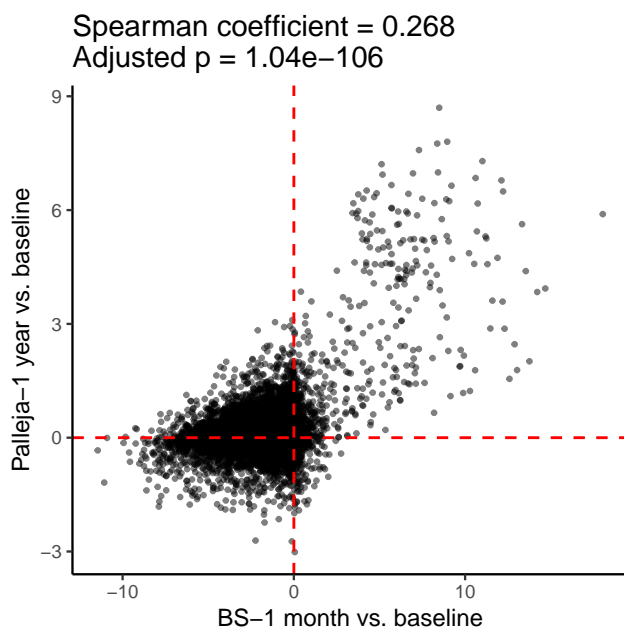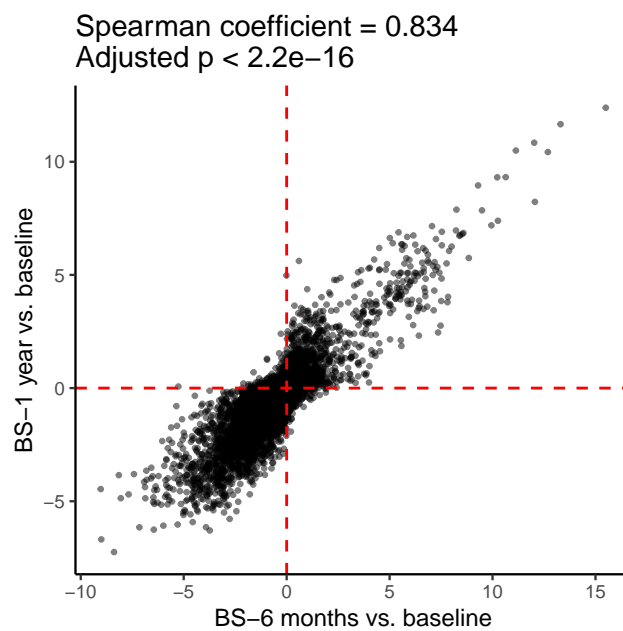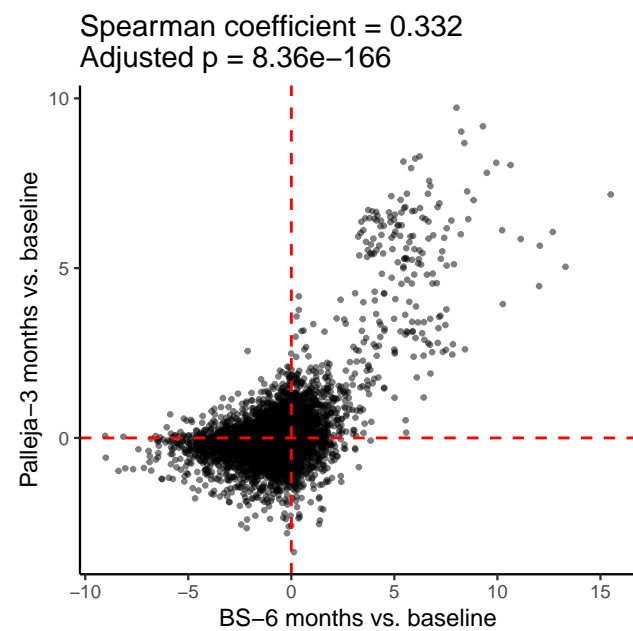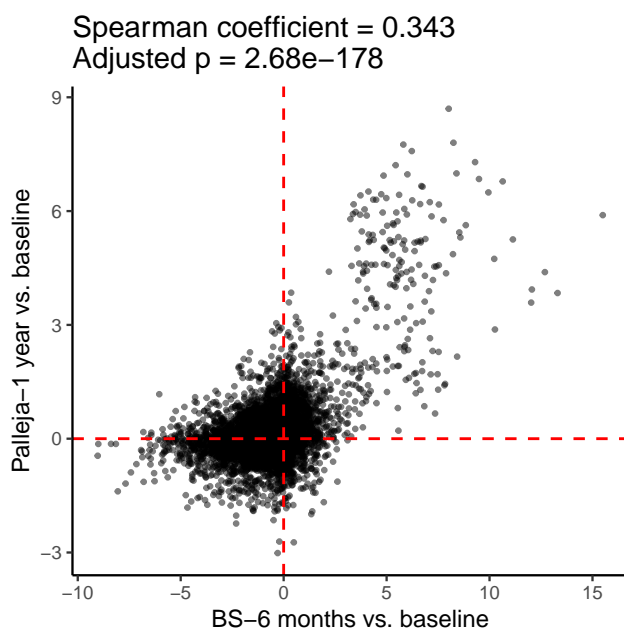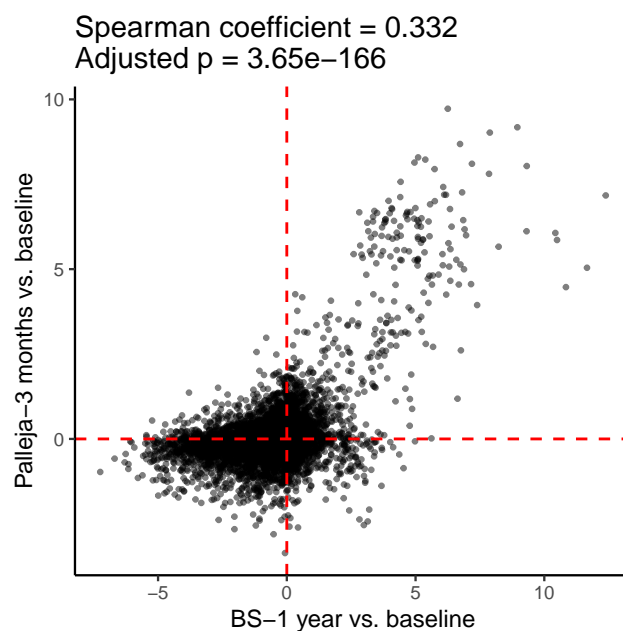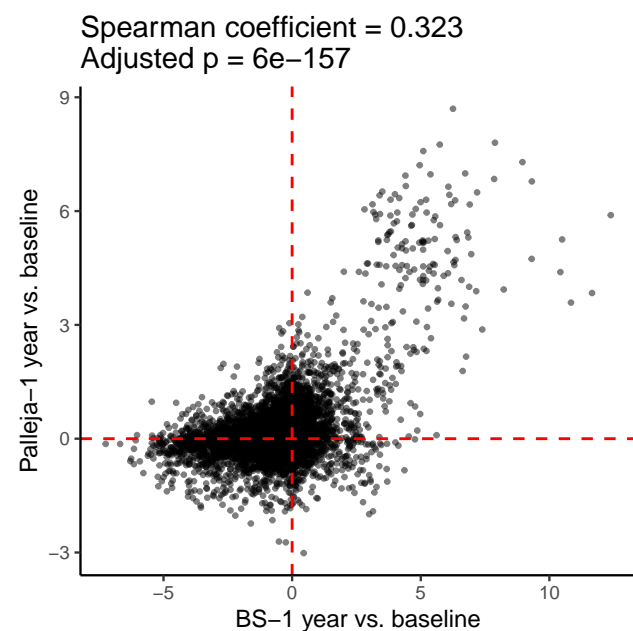

Spearman coefficient = 0.708  
Adjusted p < 2.2e-16

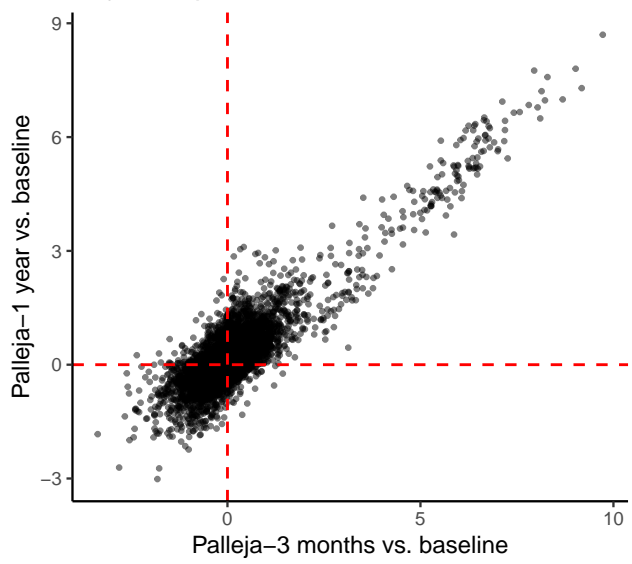

Supplement: Supplemental Material [file KGMI_A_1930872_SM8444.zip › supplementary/FIGURE 5.pdf]

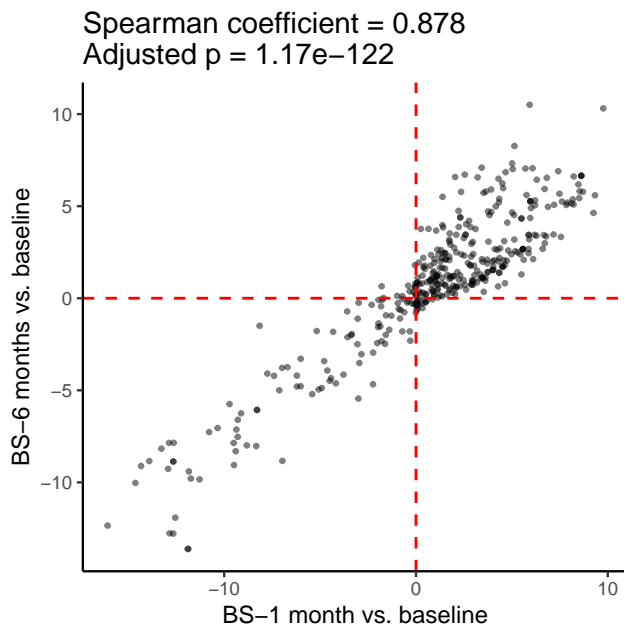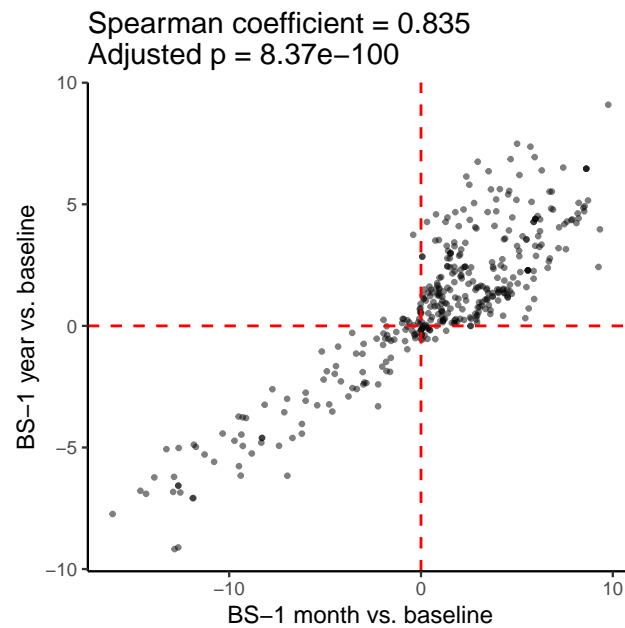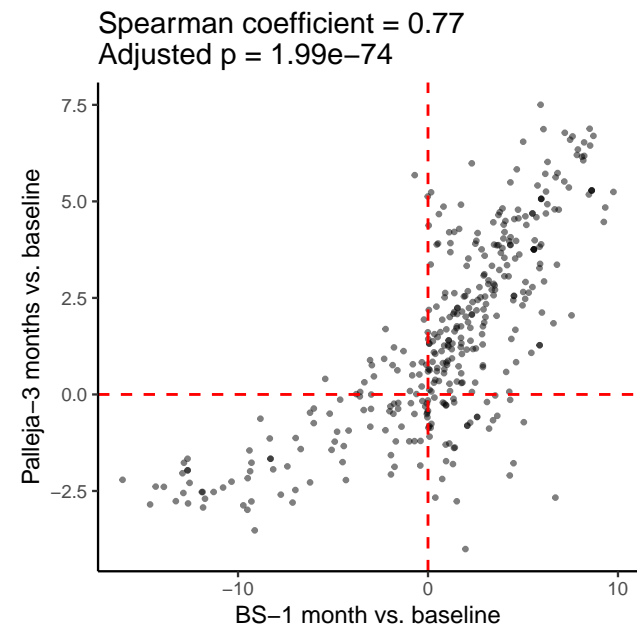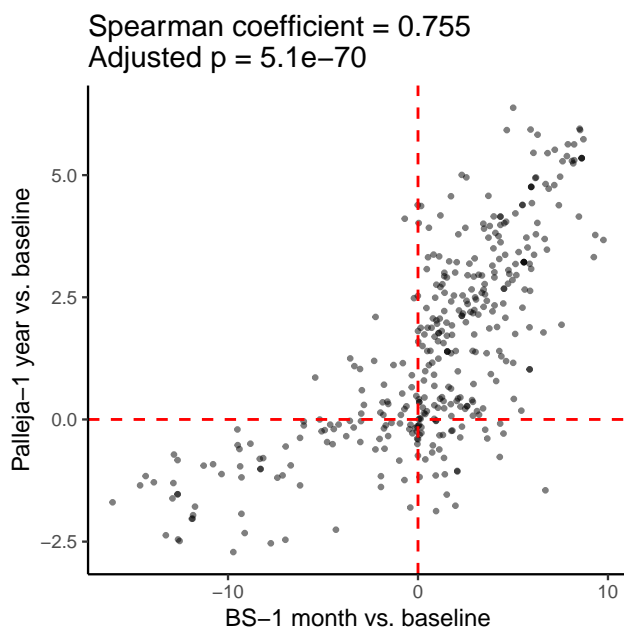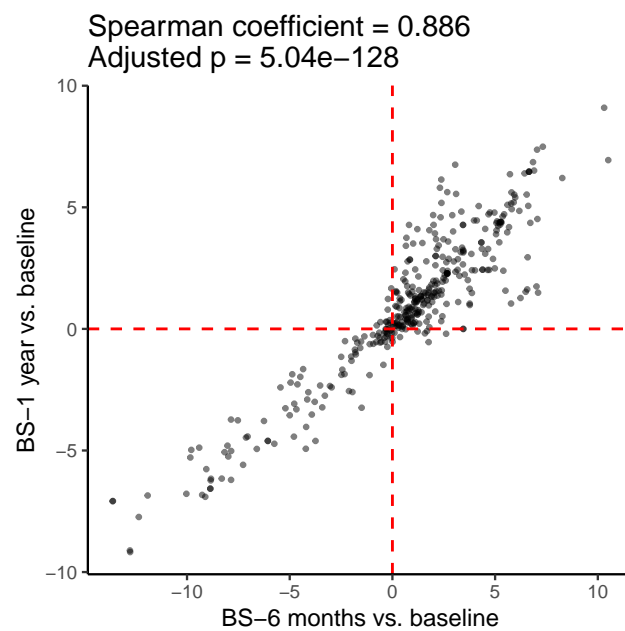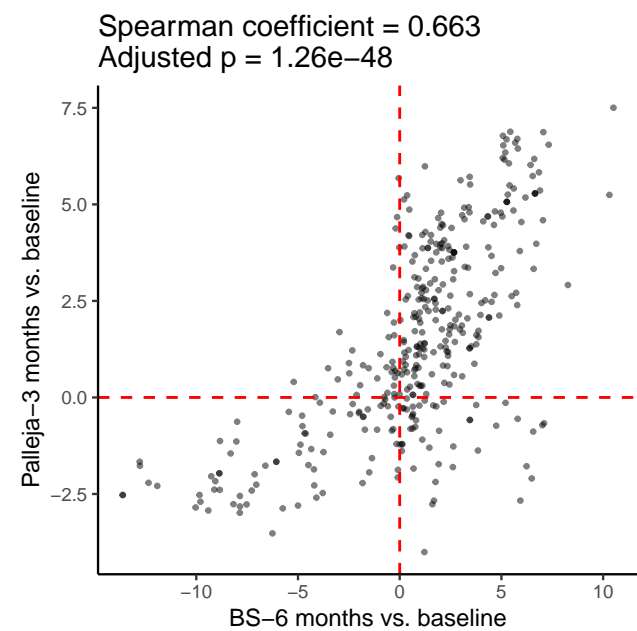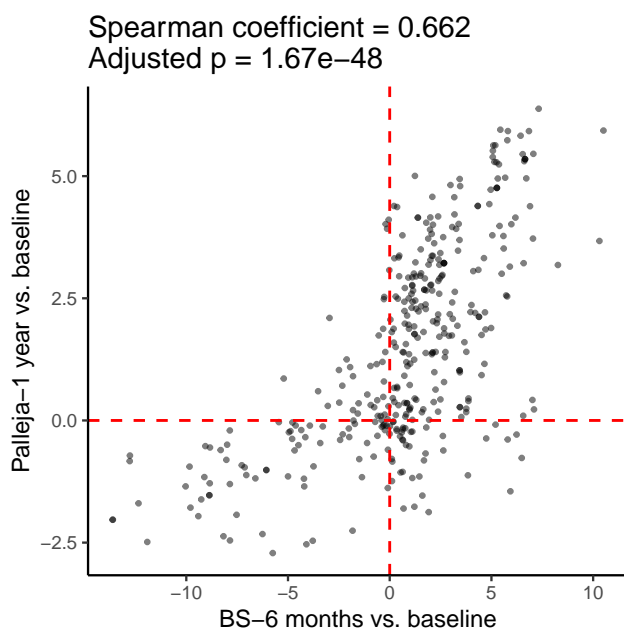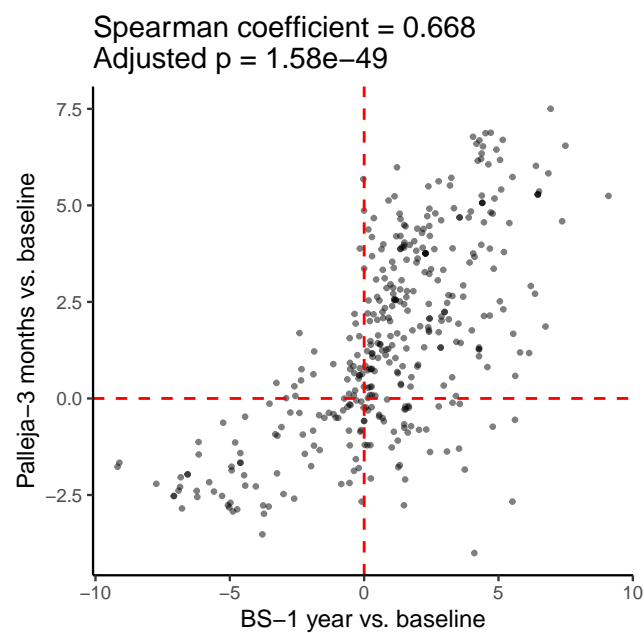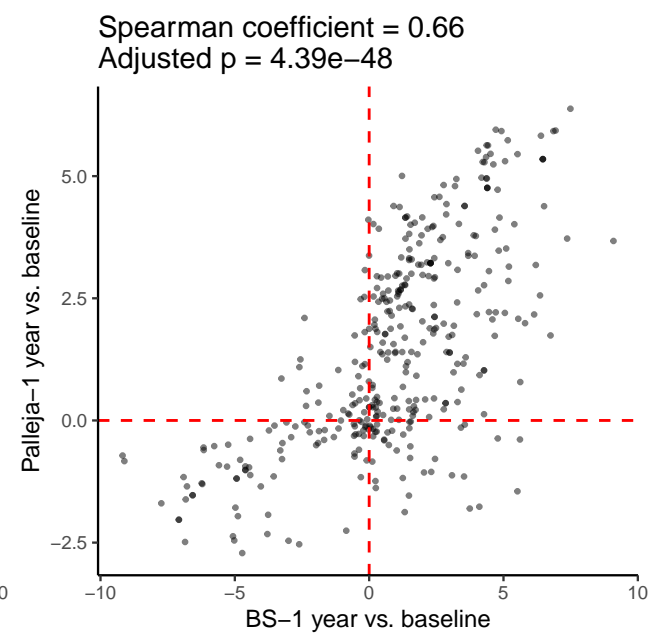

Spearman coefficient = 0.954  
Adjusted p = 1.92e-201

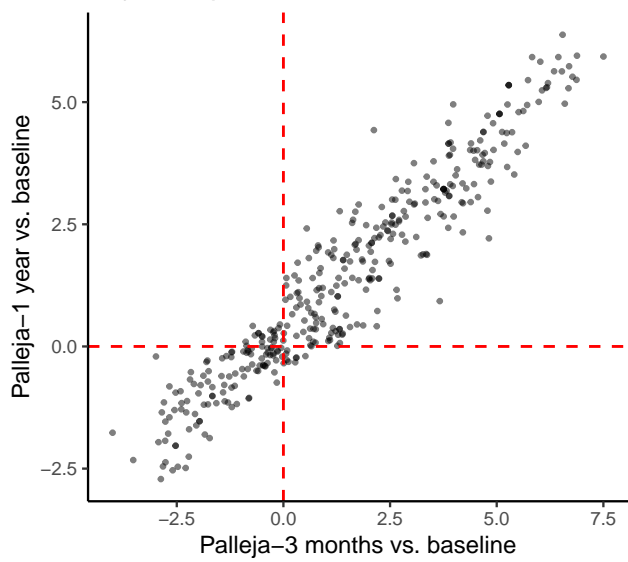

Supplement: Supplemental Material [file KGMI_A_1930872_SM8444.zip › supplementary/FIGURE 6.pdf]

A

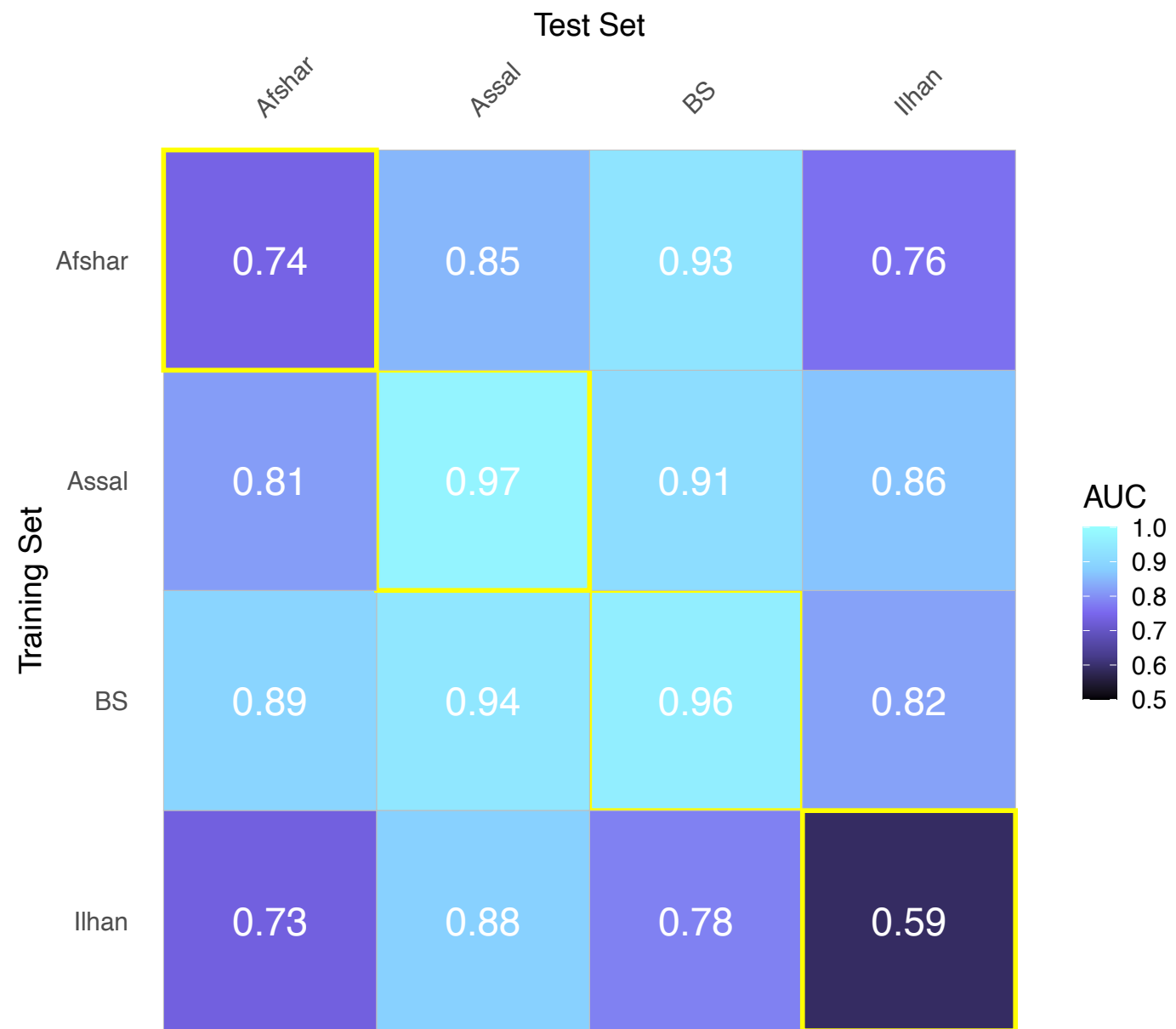

Model Average

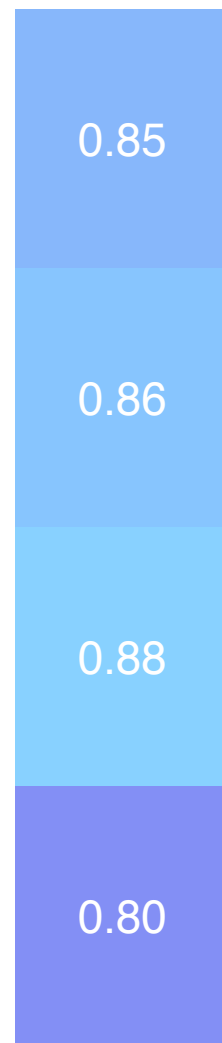

B

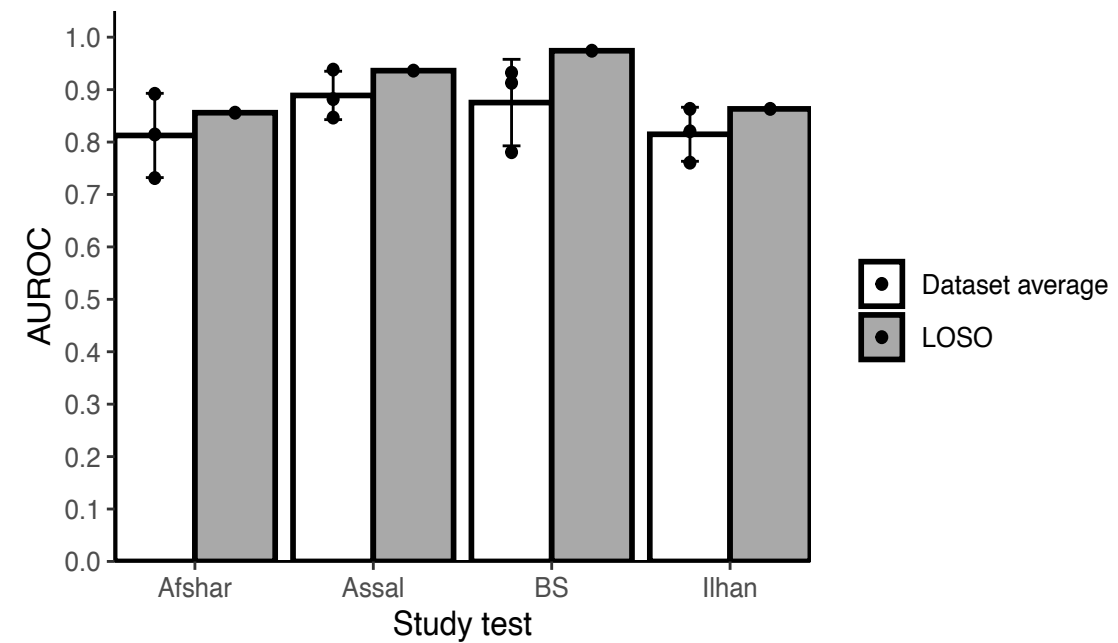

Supplement: Supplemental Material [file KGMI_A_1930872_SM8444.zip › supplementary/FIGURE 7.pdf]
